# Cisplatin as an Anti-Tumor Drug: Cellular Mechanisms of Activity, Drug Resistance and Induced Side Effects

**DOI:** 10.3390/cancers3011351

**Published:** 2011-03-15

**Authors:** Ana-Maria Florea, Dietrich Büsselberg

**Affiliations:** 1 Department of Neuropathology, Heinrich-Heine University, Düsseldorf, Germany; E-Mail: anamflorea@gmail.com; 2 Weil Cornell Medical College in Qatar, Qatar Foundation-Education City, P.O. Box 24144, Doha, Qatar

**Keywords:** cisplatin, cancer, intracellular mechanisms, ROS intracellular calcium, multi drug resistance, ROS, combinatorial therapy, toxicity, cell death, DNA damage and repair, apoptosis

## Abstract

Platinum complexes are clinically used as adjuvant therapy of cancers aiming to induce tumor cell death. Depending on cell type and concentration, cisplatin induces cytotoxicity, e.g., by interference with transcription and/or DNA replication mechanisms. Additionally, cisplatin damages tumors via induction of apoptosis, mediated by the activation of various signal transduction pathways, including calcium signaling, death receptor signaling, and the activation of mitochondrial pathways. Unfortunately, neither cytotoxicity nor apoptosis are exclusively induced in cancer cells, thus, cisplatin might also lead to diverse side-effects such as neuro- and/or renal-toxicity or bone marrow-suppression. Moreover, the binding of cisplatin to proteins and enzymes may modulate its biochemical mechanism of action. While a combination-chemotherapy with cisplatin is a cornerstone for the treatment of multiple cancers, the challenge is that cancer cells could become cisplatin-resistant. Numerous mechanisms of cisplatin resistance were described including changes in cellular uptake, drug efflux, increased detoxification, inhibition of apoptosis and increased DNA repair. To minimize cisplatin resistance, combinatorial therapies were developed and have proven more effective to defeat cancers. Thus, understanding of the biochemical mechanisms triggered by cisplatin in tumor cells may lead to the design of more efficient platinum derivates (or other drugs) and might provide new therapeutic strategies and reduce side effects.

## 1. Introduction

Metals are ubiquitous and some are essential for cellular processes. Depending on their availability, metals were selected during the process of evolution to improve biochemical processes involved in cellular processes. While metals have specific characteristics that include redox activity, variable coordination modes, and reactivity towards organic substrates, there intracellular availability is tightly regulated. Thus, aberrant metal ion concentrations are associated with various pathological disorders, including cancer. Nevertheless, metal compounds are potential candidates as anticancer agents [1].

Cisplatin is one of the most potent chemotherapy drugs widely used for cancer treatment. The discovery of cisplatin, *cis*-[Pt(II)(NH(3))(2)Cl(2)] ([PtCl_2_(NH_3_)_2_] or CDDP), was a corner stone which triggered the interest in platinum(II)- and other metal-containing compounds as potential anticancer drugs. Clinical use of CDDP determined that many patients with different types of cancer have been successfully treated, including: sarcoma cancers, cancers of soft tissue, bones, muscles, and blood vessels. Such cancers received better prognosis and therefore became less life threatening these days [2,3]. Clinical success of CDDP and its derivatives determined considerable effort to develop other effective metal-based anti-cancer compounds [1,4,5]. However, its use is limited due to side effects in normal tissues.

Beside CDDP, multiple platinum derivatives were tested in clinical trials. However, only a few (e.g., carboplatin and oxaliplatin) received worldwide approval for clinical practice, while nedaplatin, oxaliplatin, lobaplatin and heptaplatin received limited approval for clinical use. But, most other platinum compounds seem not to offer substantial clinical advantages. Therefore, the synthesis of other platinum anticancer drugs (e.g., that could form DNA adducts or target other proteins/enzymes and pathways that are critical for cytotoxicity and/or apoptosis) became the focus of recent research [3,6–8].

**Figure 1.**
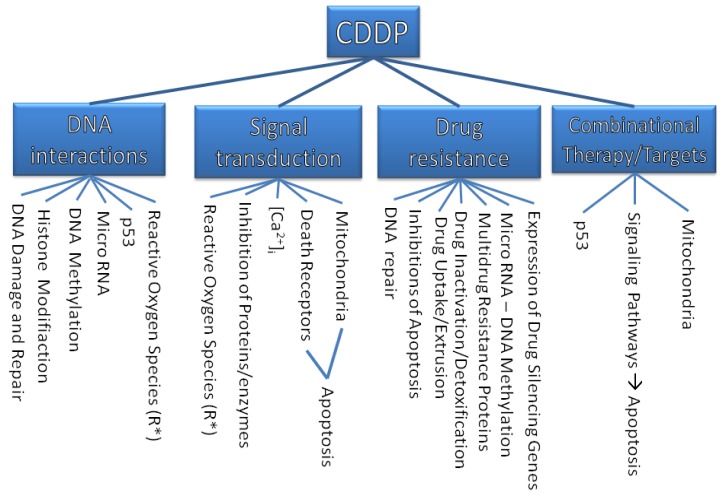
The targets of CDDP.

The purpose of this review is to describe molecular mechanisms that mediate the sensitivity of cancer cells to this drug and to show how our knowledge of some critical molecular events has been improved. This solid foundation will give rise to a more rational approach in anticancer drug design. An overview on the discussed targets of CDDP is illustrated in Figure 1.

### 1.1. Historical Background

The discovery of CDDP as an anti-cancer drug in the 1960s opened a new era in cancer treatment [5]. More than 100 years earlier, in 1845, it was synthetized for the first time and in 1893 Alfred Werner deduced the structure of CDDP [3]. In the 1960s, Rosenberg and colleagues discovered that electrolysis of a platinum electrode results in generating cisplatin. Later the same group successfully tested the effects of several platinum complexes on rat sarcomas [2].

In 1971, CDDP was applied to a cancer patient [7] for the first time. It became available for clinical practice in 1978, as Platinol® (Bristol-Myers Squibb). CDDP showed a high level and broad spectrum of antitumor activity and, therefore, extensive research developed in the area of drug design to find novel non-platinum-containing metal species with superior anti-cancer activity and low side effects [5,7,8]. CDDP has clinical benefit for several types of solid tumors; however, the efficiency of CDDP is often accompanied by toxic side effects and tumor resistance, which in turn leads to secondary malignancies [5].

The main goal of scientific research focusing on platinum complexes is to identify compounds that have superior efficacy, reduced toxicity, lack of cross-resistance or improved pharmacological characteristics as compared with the parent compound, cisplatin [3,7]. Additionally, combination therapies to treat cancer are quite common. CDDP is frequently used in combination with one, two, three, or even four other drugs, with positive results. The hope is that the drugs will work together, producing synergistic or at least additive effects in killing cancer cells, while producing no additional side effects [3].

### 1.2. Platinum in the Adjuvant Therapy of Cancers and Side Effects

Clinically, platinum complexes are used as adjuvant therapy of cancers. The aim of anticancer therapies is to induce tumor cell death. CDDP is one of the most commonly used compounds and is used for a wide spectrum of human malignancies [6,9]. Platinum compounds, are used for the treatment of a spectrum of specific cancers, including testicular, ovarian, bladder, head and neck, esophageal, small and non-small cell lung, breast, cervical, stomach and prostate cancers, as well as Hodgkin's and non-Hodgkin's lymphomas, neuroblastoma, sarcomas, multiple myeloma, melanoma, and mesothelioma.

Despite the positive effects of platinum compounds, they are poisons. Patients receiving these agents experience severe side effects that limit the dose which can be administered. The ability to manage this induced toxicity is crucial for the success of cancer chemotherapy. Side effects of platinum therapy include general cell-damaging effects, such as nausea and vomiting, decreased blood cell and platelet production in bone marrow (myelosuppresion) and decreased response to infection (immunosuppression). More specific side effects include damage to the kidney (nephrotoxicity), damage of neurons (neurotoxicity) and hearing loss [3,9–12]. Key management principles and strategies include renoprotection and enhancing drug elimination with intravenous hydration with the option of using an osmotic diuretic, while avoidance of nephrotoxic medications is necessary. Sodium thiosulfate and plasmapheresis, with or without hemodialysis support, are considered as well as the monitoring of clinical and laboratory parameters. In addition, application of additional therapies, including antiemetics and hematopoietic colony stimulating factor support, are necessary [12].

Since cisplatin-related toxicities are dose-dependent, an over dosage might result in significant morbidity and/or mortality, while the incidence of cisplatin overdose remains unknown. To date, general guidelines do not exist in order to manage a CDDP overdose. No specific antidote exists. However, early-phase clinical trials utilizing a high-dose of CDDP, and case reports in the overdose setting have characterized the clinical features associated with CDDP overdoses, highlighting some therapeutic strategies for consideration [12].

CDDP is used to treat *pediatric solid tumors*. A major dose-limiting toxicity in children is hearing loss [13]. A large individual variation exists, since it does not occur in all patients even if they are similarly treated [13]. Rademaker-Lakhai and co-workers determined the *auditory toxicity* associated with dose- and schedule- intensive cisplatin/gemcitabine chemotherapy in non-small-cell lung carcinoma patients. Hearing loss occurs mainly at high frequencies and is more pronounced when cisplatin is given once every second week [14]. Overall, there is a need for otoprotective agents that could be administered without compromising cancer treatment [15].

CDDP is *nephrotoxic* [16]. Recent evidence supports the role of CDDP for an inflammatory pathogenesis with T lymphocytes as direct mediators of (experimental) CDDP nephrotoxicity. Targeting T lymphocytes could reduce these side effects when CDDP is applied to cancer patients [17]. In rats, CDDP also induces acute renal failure and down-regulation of hepatic cytochrome P450 (P450) in an isozyme selective manner [18]. The mechanism of this enzyme inhibition appears to be a direct interaction with sulfhydryl groups [19].

*Cardio toxicity* has also been associated to CDDP treatment. Antineoplastic chemotherapy with CDDP might result in alterations of the electrocardiogram including a prolongation of QT-interval and emesis of different grades. A co-application with effective antiemetics is contraindicated since synergistic effects on the QT-interval could occur. Therefore, the knowledge of the pharmacological properties of anti-cancer drugs in general is vital to reduce risks, thereby exposing particularly vulnerable individuals, such as those with congenital long QT syndrome, to a CDDP treatment [20].

Little attention has been given to *hepatotoxicity*. Therefore, CDDP has been rarely characterized and studied as hepatotoxicant. In rats, CDDP increases lipid peroxidation and alters the thiol status of the tissue with concomitant alterations in the enzymatic antioxidants. Glutathione and glutathione reductase levels are significantly decreased after CDDP therapy, whereas glutathione peroxidase, gamma-glutamyl transpeptidase and catalase show a significant increase. Nevertheless, the glutathione-S-transferase activity was not altered by CDDP. CDDP treatment also increases the cytochrome P 450 level whereas cytochrome b(5) is decreased. Thus, an alteration in enzymatic antioxidant status with an increase in lipid peroxidation indicates that these enzymes play an important role in combating oxidative stress induced by free radicals [21].

In order to overcome the side effects of CDDP, as well as drug resistance and poor oral bioavailability, development of platinum-based drugs from carboplatin and oxaliplatin to satraplatin, picoplatin and the multinuclear platinum complex BBR3464 (triplatin) is required [3,8,9]. However, despite encouraging preclinical *in vitro* and *in vivo* results, the outcome of clinical trials of the new platinum compounds remained below expectations. Possible explanations include: possible activation of “active species” generated *in vivo*, as well as uptake, efficient efflux, or intracellular trafficking and other possible mechanisms involved in intrinsic and acquired chemo resistance of platinum drugs *in vivo*, including the insufficient diffusion in tumor tissues that could also constitute an important limiting step in the treatment of solid tumors with platinum compounds.

Preclinical assays with improved predictive power for the clinical outcome of the compounds are currently underway and are based on representative tumor models, such as resistant primary cancer cell lines, spheroids and orthotopic xenograft models, respectively [8,9]. Nevertheless, the use of new drugs and drug combinations (improved pharmaceutical formulations, bifunctional complexes), as well as the use of decisive preclinical selection tests, will hopefully result in the generation of more efficient platinum-based anticancer drugs that will show clinical activity even in multidrug-resistant tumors [8]. The research concentrated on platinum analogues focuses on two strategies: expanding the utilization of platinum therapy to other tumor types not usually treated with platinum compounds, while another area of research represents novel routes of administration [7].

Although current treatment protocols including surgery and chemotherapy achieve local control of the tumor, patients could relapse and further progress to develop a systemic disease. Randomized trials of CDDP-based chemotherapy regimens in the neoadjuvant setting demonstrated potential to improve survival. With the introduction of new cytotoxic drugs and novel small molecules (e.g., siRNA), there is a need to conduct studies to find optimal use of therapy in high-risk cancer patients [22,23].

## 2. Cellular Activity of Platinum Compounds

In recent years, the focus of medical research was to elucidate the mechanisms underlying cancer. The development of new techniques that identify perturbations of cell functions resulted in an increased knowledge in molecular, physiological and pathological mechanisms of cancer. Prior to the understanding of the mechanisms of cancer, chemotherapeutic agents were discovered empirically, basically inhibiting metabolic pathways necessary for cell division (e.g., DNA damage). However, none of these anticancer strategies are particularly specific for cancer [3]. Nevertheless, cancers are complex. Thus, different cancers have different features; therefore, it is unlikely to find one cure to treat all cancers by targeting only one specific gene product or one pathway that is required for tumor growth [24]. For instance, cancer cells are non-apoptotic [25] and chemotherapy affects rapidly growing normal, as well as, cancer cells [25]. Overall, a better understanding of the molecular mechanisms behind the modulation of cellular responses is needed to develop and optimize new therapeutic strategies for the use of CDDP [26].

**Figure 2.**
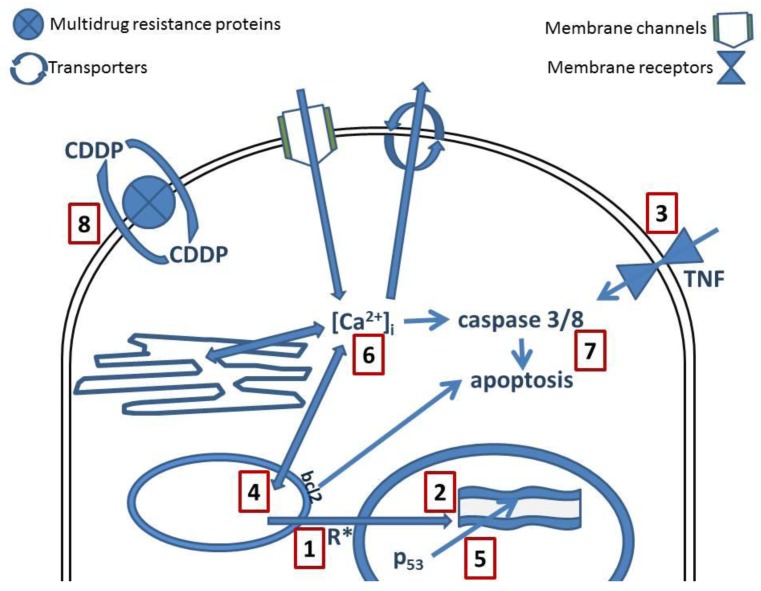
Cellular interactions of CDDP: (**1**) reactive oxygen species; (**2**) DNA; (**3**) TNF; (**4**) mitochondria; (**5**) p53; (**6**) calcium signaling; (**7**) caspases; (**8**) multidrug resistant proteins.

Molecular and genetic approaches have enhanced the understanding of cell biology and they have uncovered new signaling networks that regulate proliferation and survival. Some of these networks are altered in cancer cells. Platinum compounds damage tumors via induction of apoptosis, which is mediated by the activation of various signal transduction pathways (Figure 2). It is discussed that this effect is related to inhibition of DNA synthesis and repair that might result in cell cycle arrest at the G1, S, or G2-M phase, therefore apoptosis will be induced [3].

### 2.1. Reactive Oxygen Species

Exposure to oxidative stress can disrupt normal biological functions (Figure 1). Apart from DNA damage, recent data suggest that CDDP also induces reactive oxygen species (ROS) that trigger cell death (Figure 2(1)). Cell death occurs upon simultaneous activation of several signaling pathways, while the specific pathways depend on the (cancer) cell. The formation of ROS depends on the concentration of CDDP and the duration of exposure [26]. The intracellular redox homeostasis is maintained by the thiol group (-SH) containing molecules. Under certain conditions a thiol group may lead to formation of thiyl radicals that in turn can interact with molecular oxygen, therefore generating ROS [27].

Most metals are able to generate ROS. Several DNA damages are mediated by metal-induced free radicals, while in addition metals can inhibit the repair of DNA. Multiple mechanisms result in repair inhibition: (a) directly by free radical, substitution of zinc in Zn-finger domains; or (b) indirectly by lowering the level of reduced glutathione [27]. This might partially explain the paradox that ROS might be carcinogenic, but also useful to treat cancer. These different effects might occur due to different concentrations and time of exposure. Metal concentrations exceeding specific physiological levels are toxic, possibly by generating free radicals. Usually, each cell has a balance between antioxidants and free radicals. While ROS might overwhelm the reduction capacities of the cell; they induce lipid peroxidation, depletion of the sulfhydryl groups, altered signal transduction pathways, calcium homeostasis and DNA damage. This consequently results in ageing and/or cancer [27].

Although recent evidence underlines the importance of CDDP-induced ROS *in vitro*, similar data are still not available for primary tumor tissue. Some reports show that CDDP induces ROS formation *in vivo*, which is responsible for the severe side effects of CDDP therapy, including nephrotoxicity and hepatotoxicity, which in turn, are reduced by the addition of antioxidants. Nevertheless, understanding the expression of anti- and pro-apoptotic proteins and their relationship to redox system in the cell, as well as the location of ROS generation upon CDDP treatment will provide valuable insight into new approaches for the prevention of CDDP side effects without affecting its efficacy [26].

### 2.2. Cytotoxicity

Although it is still debatable whether the clinical success of CDDP relies primarily on its ability to trigger apoptosis, at least two distinct pathways have been proposed to contribute to cisplatin-induced apoptosis *in vitro*. One involves the tumor-suppressor protein p53, the other is mediated by the p53-related protein p73. Coupling cisplatin damage to apoptosis requires mismatch repair activity, and recent observations further suggest an involvement of the homologous re-combinatorial repair system. At present it is generally accepted that abortive attempts to repair the DNA lesions play a key role in the cytotoxicity of the drug, and loss of the mismatch repair activity is known to cause cisplatin resistance, a major problem in antineoplastic therapy [28].

#### 2.2.1. Interaction with the DNA: DNA damage and DNA repair mechanisms

CDDP is primarily considered as a DNA-damaging anticancer drug, forming different types of bifunctional adducts with cellular DNA (Figure 2(2)) [4,26,29] while the XRCC1 gene may have significant impact on the response of patients to platinum-based chemotherapy [30]. While cisplatin and oxaliplatin are structurally distinct but they form similar adducts at the same sites of the DNA [31–33]. Such adducts are recognized by a number of cellular proteins. For example, mismatch repair proteins and some damage-recognition proteins bind to cisplatin-GG adducts with higher affinity than to oxaliplatin-GG adducts. This differential recognition of cisplatin- and oxaliplatin-GG adducts contribute to the differences in cytotoxicity and tumor range of CDDP and oxaliplatin [31]. Nevertheless, there might be significant conformational differences between adducts, even if the crystal structures of CDDP- and oxaliplatin-GG adducts are similar, e.g., cisplatin could cause a disruption of cellular activity through binding to a more sensitive region of the genome, such as the telomeres [29]. On the other hand, there is also the possibility that platinum drugs might interact directly with free nucleobases before their incorporation in DNA [34].

The research of new platinum drugs active towards CDDP resistant tumors has been mostly focused on compounds with *cis*- geometry because *transplatin*, the *trans*-isomer of cisplatin, is inactive. Transplatin's inactivity is the result of kinetic instability promoting its deactivation and the formation of DNA adducts characterized by a region-selectivity and a stereochemistry different from cisplatin. Therefore, it is speculated that the presence of two groups in *cis*- positions are necessary for antitumor activity of platinum complexes [35]. However, at least several exceptions have been reported: substitution of transplatin ammine ligands by aromatic N-donor heterocycles resulted in aliphatic amines, or imino ligands, that gave compounds with the characteristic of inhibiting tumor cell growth, and was active against cisplatin resistant tumor cells [35]. Nevertheless, the formation of DNA adducts that are qualitatively and quantitatively different from CDDP supports the hypothesis that antitumor-active trans-platinum complexes might have a different spectrum of activity [35].

*Oxaliplatin* (Eloxatine) is a third-generation platinum compound that shows *in vitro* and *in vivo* anti-tumor effects. In addition, it has a better safety compared to CDDP and no cross-resistance with CDDP and carboplatin. Oxaliplatin has a non-hydrolysable diaminocyclohexane carrier ligand, which is maintained in the final cytotoxic metabolites of the drug [36]. Like CDDP, oxaliplatin targets DNA producing mainly 1,2-GG intrastrand cross-links. The cellular and molecular aspects of the mechanism of action of oxaliplatin are not fully understood. Nevertheless, mismatch repair and replicative bypass appear to be the processes most likely involved in differentiating the molecular responses to these agents [36].

### 2.3. Extrinsic Apoptosis

Cancer therapy aiming at a direct induction of cell death by activation of the *tumor necrosis factor-related apoptosis-inducing ligand* (TRAIL) receptor-mediated signal-transduction pathways is an important variant for antitumor strategies. In fact, it has entered clinical development. Malignant cells are frequently refractory to the cytotoxic effect of this apoptosis-inducing ligand, despite the fact that they adequately express TRAIL receptors DR4/DR5. The susceptibility of cancer cells to TRAIL can be increased by CDDP [37].

*Tumor necrosis factor* (TNF)-related apoptosis-inducing ligand (TRAIL/Apo2L) is a member of the TNF ligand family (Figure 2(3)). TRAIL is normally expressed in the human immune system and plays a critical role in antitumor immunity. It selectively induces apoptosis in tumor cells *in vitro* and *in vivo* but not most normal cells [37,38]. TRAIL interacts with the death receptors, DR4 and DR5, and activates intracellular apoptotic pathways. Binding of TRAIL to its receptors DR4 and/or DR5 results in receptor aggregation, recruitment of Fass-associated death domain, procaspases 8 (and 10) (and other adaptor molecules) to form the death-inducing signaling complex that lead to activation of caspases (Figure 2(7)). The activated caspase 8 can cleave and activate caspases 3 and 7, leading to apoptosis via the extrinsic pathway, in contrast to the mitochondria-mediated intrinsic death signaling pathway activated following cytotoxic stresses [37,38]. The discovery that TRAIL activates intracellular apoptotic pathways in cancer cells has resulted in development of anticancer drugs that selectively activate these pathways. These therapeutic agents include recombinant human TRAIL and its agonistic monoclonal antibody against DR4 and DR5. Phase I trials have established the safety and tolerability of these TRAIL agonists in patients. Phase II trials are evaluating the therapeutic efficacy of TRAIL agonists as single agents or in combination with established cancer therapeutics [39]. Studies have also shown that TRAIL-resistant renal cell carcinoma, prostate gland cancer, and bladder cancer cells, are sensitive to concentrations of chemotherapeutic drugs (including doxorubicin, epirubicin, pirarubicin, and cisplatin), which are below their toxic concentrations. Therefore, TRAIL, particularly in combination with chemotherapeutic agents, is a potentially promising substance in the treatment of cancer [40].

### 2.4. Intrinsic Apoptosis

#### 2.4.1. Mitochondria

Mitochondria are the powerhouse of the cell, providing energy. The malfunction of mitochondria has implications in the pathogenesis of a variety of disorders, including cancer and multiple neurodegenerative diseases. Disturbance of mitochondrial vital functions, e.g., production of ATP, calcium buffering capacity, and generation of reactive oxygen species, might be involved in disease pathogenesis. Mitochondria are a source of important mediators of apoptosis (Figure 2(4)).

Mitochondria have recently emerged as targets for anticancer drugs. The specific bioenergetics' features of mitochondria have been used in the design of pharmacological agents. A variety of compounds were identified that act by interfering with the function of mitochondria. These compounds destabilize mitochondria and cause apoptosis selectively in cancer cells [24]. Drugs that intentionally change the function of mitochondria are the choice for a number of different types of the neoplastic disease [24]. Alterations in the mitochondrial function of tumor cells might lead to cell survival and therefore in the resistance of tumor cells to chemotherapy.

The permeabilization of mitochondrial membranes is associated with apoptosis. Mitochondrial membrane permeabilization (MMP) may occur via the control of proapoptotic Bcl-2 family members, and/or by the induction of the mitochondrial permeability transition. These cell death-inducing regulatory mechanisms are ultimately responsive to the bioenergetic status/redox state of mitochondria. In addition to inducing MMP, chemopreventive agents might directly modulate mitochondrial bioenergetics and/or the redox state that might be important targets in the chemoprevention of cancer [41].

However there is only little information available on how cisplatin interacts with mitochanodria. It is discussed whether the relation between the mitochondrial density in the cells and the cellular sensitivity to the toxicity of CDDP is a key factor for the determination of the anticancer activity and side effects of CDDP [42]. One example is given by the study of Muscella *et al.*, 2008. They studied the effects of cisplatin and [Pt(O,O′-acac)(gamma-acac)(DMS)] in MCF-7 cells which have been proven resistant to many chemotherapeutics. Compared with cisplatin, the cytotoxicity of [Pt(O,O′-acac)(gamma-acac)(DMS)] correlated with cellular accumulation but not with DNA binding. The second compound triggered apoptotic processes in MCF-7 cells implicating mitochondria depolarization; cytochrome c accumulation in the cytosol; translocation of Bax and truncated-Bid from cytosol to mitochondria and decreased expression of Bcl-2; cleavage of caspases -7 and -9, and PARP degradation; chromatin condensation and DNA fragmentation [43]. However, mitochondria could also be unintended offside targets of other drug therapies and thereby responsible, at least in part, for adverse events associated with many treatments [44]. Overall, targeting of mitochondria can be beneficial in the treatment of cancers [45].

#### 2.4.2. The tumor suppressor protein p53

Mutations in the TP53 tumor suppressor gene are the most frequent genetic alteration in human cancers. These alterations are mostly no-sense point mutations that cluster in the DNA binding domain (Figure 2(5)). Multiple of these mutations generate mutant p53 proteins that acquire new biochemical and biological properties that might contribute to tumor malignancy. The inhibition of the mutant p53 expression by RNA interference of endogenously expressing mutant p53 proteins reduces cell proliferation, *in vitro* and *in vivo* tumorigenicity, and resistance to anticancer drugs. Conclusively, knocking down the mutant p53 weakens human cancer cells [46].

The p53 tumor repressor protein is related to the sensitivity to CDDP in cancer [47,48]. Cisplatin-inducible p53 gene therapy might provide a control of transgene expression while enhancing the effectiveness of commonly used chemotherapeutic agents. This might be a promising new treatment for cancers [49]. Such an example could be represented by the study of Park and co-workers who investigated the relationship of the p53 status and chemo-sensitivity to cisplatin in two human malignant glioblastoma cell lines (A172 and T98G, harboring wild-type and mutant-type p53, respectively). The authors concluded that the expression of p53 is essential for the cytotoxic effect of CDDP and the introduction of apoptotic signal molecules, such as p53, will be beneficial to achieve chemosensitivity in malignant glioma [47].

The p53 protein can recognize DNA modified with CDDP. Pivonkova and colleagues investigated the p53 binding to the CDDP-DNA using p53 deletion mutants and via modulation of the p53-DNA binding by changes of the protein redox state. Isolated p53 C-terminal domain bound to the CDDP-DNA with a significantly higher affinity than to the unmodified DNA [50].

Protein kinase C (PKC) *δ* is a positive regulator for CDDP-induced cell death. In a study by Kager and co-workers, they examined whether over-expression of PKCδ and p53 increases the sensitivity of the human gastric cancer cell line, MKN28, which has a mutation of p53 gene, to CDDP. It was concluded that PKCδ, in cooperation with p53, possibly regulates CDDP-induced caspase-3-mediated cell death in gastric cancer [48].

#### 2.4.3. Epigenetic interventions

There is less evidence concerning epigenetic modifications induced by CDDP. Histone modifications and DNA methylation are epigenetic phenomena that play a critical role in many neoplastic processes, including silencing of tumor suppressor genes. One such histone modification, particularly at H3 and H4, is methylation at specific lysine (K) residues, whereas histone methylation of H3-K9 is linked to DNA methylation and aberrant gene silencing in cancer cells [51].

*Post-translational modification of histones* alters chromatin structure, facilitating the binding of nuclear factors that mediate DNA repair, transcription, and other processes. The effects of CDDP treatment on histone post-translational modification in cancer cells were studied and specific phosphorylation of histone H3 at Ser-10, mediated by the p38 MAPK pathway, were induced in response to CDDP treatment. In addition, hyperacetylation of histone H4 was caused by drug treatment. These findings revealed a link between CDDP administration and chromosomal structural alterations, providing mechanistic information about how cells respond to platinum-induced stress [52]. Interestingly, histone deacetylase inhibitors are anticancer agents that act by up-regulating the expression of cell-cycle control genes. The histone deacetylase inhibitor (sodium butyrate) gives protection in a single-dose model of CDDP ototoxicity in guinea pigs. Because histone deacetylase inhibitors are anticancer agents with very few side effects, they may be candidates for clinical use during CDDP chemotherapy [15].

*MicroRNAs* are small noncoding RNAs that are produced endogenously and have emerged as important regulators in pathophysiological conditions, such as tumorgenesis, as well as development [53]. To explore the mechanism of apoptosis induced by cisplatin, the expression of microRNAs (miRNAs) and regulating genes in K562 cells was analyzed by Xie *et al.*, using reverse transcription PCR, quantitative real-time PCR and enzyme-linked immunosorbent assays. MiR-16, miR-34a-c, miR-17-5p and miR-125 were up-regulated, while their associated oncogenes (BCL2, E2F1 and E2F3, respectively) were down-regulated after CDDP treatment. In addition miR-106 and miR-150 were down-regulated while their target genes (RB1 and P53, respectively) were up-regulated after cisplatin treatment. Moreover, miR-16, miR-34a-c and miR-17-5p proved to be upstream factors, regulating the expression of BCL2, E2F1 and E2F3, respectively. Their study demonstrates that CDDP induces apoptosis by reducing miR-106, which either up-regulates RB1 or inhibits miR-150 which increases P53 expression [54].

The regulation of microRNA-34a (miR-34a) was investigated by Bhatt and colleagues (2010) in an experimental model of cisplatin-induced nephrotoxicity. They observed the induction of miR-34a *in vitro* during CDDP treatment of mouse proximal tubular cells and also *in vivo* during CDDP nephrotoxicity in C57BL/6 mice. During CDDP treatment, p53 was activated. *In vivo*, miR-34a induction by CDDP was abrogated in p53-deficient mice, a result that further confirms a role for p53 in miR-34a induction during cisplatin nephrotoxicity. Functionally, antagonism of miR-34a with specific antisense oligonucleotides increased cell death during CDDP treatment. The authors conclude that, miR-34a is induced via p53 during CDDP nephrotoxicity and may play a cytoprotective role for cell survival [53].

#### 2.4.4. Calcium signaling

Recently interest has been focused on the role played by the second messenger calcium (Ca^2+^) in the physiology and pathology of cancer. Intracellular calcium ([Ca^2+^]_i_) is strictly regulated (Figure 2(6)) by a delicate balance between calcium entry and active mechanisms which transport the calcium against its concentration gradient. The regulation takes place by calcium entry from the extracellular space to induce the *increase* in [Ca^2+^]_i_ or Ca^2+^ could be released from intracellular calcium stores. The *decrease* of [Ca^2+^]_i_ is established by pumps or exchangers located at the plasma membrane at the outer cellular membrane; or through re-entry to the calcium stores, represented mainly by mitochondria and endoplasmic reticulum, via Ca^2+^-transport systems. Intracellular calcium could also be bound by calcium-buffering proteins that can further modulate [Ca^2+^]_i_. One of the most important events resulting from [Ca^2+^]_i_ signaling is the activation of biological pathways that are modulated by the binding of calcium to calcium-sensor proteins. It has been demonstrated that CDDP interferes with calcium homeostasis in different *in vitro* models [11,55,56]. CDDP increases [Ca^2+^]_i_ concentration-dependently in HeLa-S3, but not in U2-OS cells. This elevation of [Ca^2+^]_i_ depended on extracellular Ca^2+^ and not by a Ca^2+^ release triggered by Ca^2+^ entry. Nevertheless, the increase of [Ca^2+^]_i_ was reduced by the IP_3_ receptor blocker, 2-APB. Immunostaining showed IP_3_-receptors (type 1-3) at the cellular membrane of HeLa-S3 cells, but not in U2-OS cells. Electrophysiological experiments showed an increase of membrane conductance with CDDP only when Ca^2+^ was present extracellular. Increase of [Ca^2+^]_i_ was related to the activation of calpain but not caspase-8 and triggered apoptosis in HeLa-S3 but not in U2-OS cells. In small dorsal root ganglion neurons, CDDP surprisingly reduces the currents through voltage activated calcium channels in a concentration-dependent manner (1–100 μM) with an IC_50_ value of about 24 μM. Large dorsal root ganglion neurons were less sensitive [56].

PLC-mediated [Ca^2+^]_i_ signaling plays a role in CDDP resistance *in vitro* and has been proposed as a promising target to overcome CDDP resistance [57]. The activation of the PI3K/Akt pathway is a crucial step leading to angiogenesis. Consequently, the effect of Ins(1,3,4,5,6)P5 on basic fibroblast growth factor (FGF-2)-induced Akt phosphorylation, cell survival and motility was investigated and the phosphatidylinositol 3-kinase (PI3K)/Akt inhibitor Inositol(1,3,4,5,6) pentakisphosphate [Ins(1,3,4,5,6)P5] was tested. It was found that the Akt inhibitor Ins(1,3,4,5,6)P5 is a specific antiangiogenic and antitumor factor. Therefore, inappropriate activation of the PI3K/Akt pathway could be linked to the development of several diseases, including cancer, making this pathway an attractive target for therapeutic strategies. Therefore, Ins(1,3,4,5,6)P5, a water-soluble, natural compound with specific pro-apoptotic and antiangiogenic properties, might be a potential anticancer therapeutic strategy [58].

## 3. Combinatorial Therapy

Even if considerable improvement in cancer treatment has been achieved over the last decades, there are still numerous challenges. One major area of interest is focused on tumors that are characterized by relative resistance to chemotherapy and poor prognosis. To overcome drug resistance combinatorial therapies (using of 2-3 anticancer drugs with different mechanisms of actions) were developed and are successfully used in clinical practice. Also CDDP is currently used in combination with other antineoplastic drugs in *in vitro* cancer models, in chemotherapy, and is in Phase I to III clinical trials [59]. The same is basically true for oxaliplatin: its anti-tumor effects are enhanced if administered in combination with other anticancer agents, such as 5-fluorouracil, gemcitabine, cisplatin, or carboplatin; topoisomerase I inhibitors; and taxanes [60,61].

As for the single drug therapy, one major target for combinatorial therapy is also *p53*, which is often not functional or/and mutated in cancer cells. Therefore, activation of p53 or restoration of wild type p53 function provides a basis to treat human cancers [62]. PUMA, a p53 up-regulated modulator of apoptosis is a potent proapoptotic molecule that is rapidly induced in cells following DNA damage and is required for p53-induced apoptosis. Combined treatment with PUMA and anticancer drug (cisplatin, paclitaxel, 5-fluorouracil, respectively) showed that PUMA significantly increased the chemo sensitivity of esophageal cancer cells, which may result in increased apoptosis [49]. In addition, studies with adenoviral-mediated p53 gene transfer have been conducted in many cancer types including cervical, ovarian, prostatic and head and neck tumors. Using three viral constructs containing cDNA for p53 the effect of adenoviral-mediated gene therapy in four osteosarcoma cell lines demonstrated that Ad-wtp53 significantly increases sensitivity of the cell lines to CDDP and doxorubicin, these chemotherapeutic agents are commonly used in the treatment of osteosarcoma [62].

*Mitochondria* are not only the target for single drug therapies but also for the combination administration of anti-cancer agents. It has been postulated that bcl-2 has anti apoptotic and chemo protective effects and, in combination with CDDP, represents a relevant target for combinatorial therapies [63]. A study of Basma *et al.*, (Figure 2(7)) shows that the effects of delivering bcl-2 antisense and CDDP combination therapy in breast carcinoma cells p53(+)MCF-7 and p53(−)MCF-7/E6 significantly increased, apoptosis, and cytochrome c release in MCF-7/E6 than in MCF-7, but did not affect Fas levels and activated caspase-8. CDDP exerted increased apoptosis with release of cytochrome c while activating more caspase-8. However, combinatorial treatment showed improved effects on cell viability, apoptosis, cytochrome c release, and caspase-8 activation compared to individual treatments [64].

Another combinatorial therapy target could be the activation of signal transduction pathways that lead to *increased apoptosis* of the tumors cells. CDDP induces activation of N-terminal-c-Jun kinase (JNK) that, in turn, mediates induction of apoptosis [65] while combination therapy with CDDP and a PI3K inhibitor would increase the therapeutic efficacy of CDDP [66]. A new interest has been expressed in calcium signaling as a potential target for combinatorial therapy. A study of Günes and co-workers revealed that combinations of CDDP and As_2_O_3_ (each of them modulates the intracellular calcium concentration by a different mechanism: As_2_O_3_ depletes intracellular calcium stores while CDDP triggers an influx of Ca^2+^) resulted in a synergistic increase of the [Ca^2+^]_i_-signal which resulted in apoptosis. The effect was clearly larger than the additive effects of each drug alone, which could improve the anti-tumor effects [10,67,68].

## 4. Drug Resistance Mechanisms

Drug resistance is a major complication in cancer chemotherapy and accounts for the failure of chemotherapy to cure the majority of cancer patients [33,69]. Resistance occurs when tumor cells do not respond to treatment with anticancer drugs. It exists in two forms: *acquired resistance*, where the drug is initially efficient but becomes ineffective over time while *intrinsic resistance* occurs when a drug is ineffective from the beginning of the treatment. Even though CDDP has proven to be a highly effective chemotherapeutic agent for treating various types of cancers the risk of acquired drug resistance could occur. When cells become resistant to CDDP, the doses must be increased; but a large dose escalation can lead to severe multi organ toxicities [33].

Several mechanisms are involved in CDDP resistance (Figure 2(8)). Such mechanisms include decreased intracellular drug accumulation and/or increased drug efflux, drug inactivation by increased levels of cellular thiols, alterations in drug target, processing of drug-induced damage by increased nucleotide excision-repair activity and decreased mismatch-repair activity and evasion of apoptosis [29,33,70].

Additionally, there are molecular events modulating the amount of drug-DNA interaction, such as a reduction in CDDP accumulation inside cancer cells or inactivation of CDDP by thiol-containing species as well as the increase in adducts repair and a decrease in induction of apoptosis. Recently, accumulating evidence suggests a role of the long patch DNA mismatch repair system in sensing CDDP-damaged DNA and in triggering cell death through a c-Abl- and p73-dependent cascade; two other important pathways have been shown, *i.e.*, the mitogen-activated protein kinase cascade and the tumor suppressor p53 [29]. If CDDP cannot accumulate in the cell, it does not bind the DNA and cause cell death. For survival the cancer cell develops mechanisms either to keep cisplatin out of the cell or to remove cisplatin from the cell. Indeed, reducing cisplatin accumulation by cancer cells seems to be a major form of acquired resistance [33,67,69].

Cellular mechanisms of resistance to cisplatin are multifactorial and result in severe limitations in clinical use [29,69]. In general, the molecules responsible for drug resistance mechanism are up-regulated in CDDP-resistant cells; this indicates that the transcription factors activated in response to CDDP play a crucial role in drug resistance [29,69,70]. Not only the defense mechanisms might be over-expressed, but also alterations in cellular response to the drug-induced genotoxic stress might be triggered [71].

It is known that the tumor-suppressor proteins p53 and p73, and the oncoprotein c-Myc, which function as transcription factors, change the cellular sensitivity to CDDP. Several transcription factors involved in CDDP resistance have been identified, including Y-box binding protein-1 (YB-1), CCAAT-binding transcription factor 2 (CTF2), activating transcription factor 4 (ATF4), zinc-finger factor 143 (ZNF143) and mitochondrial transcription factor A (mtTFA). Two of these-YB-1 and ZNF143-lack the high-mobility group (HMG) domain and can bind preferentially to CDDP-modified DNA in addition to HMG domain proteins or DNA repair proteins, indicating that these transcription factors may also participate in DNA repair [69].

Alterations in the expression of oncogenes (such as *c-fos*, *c-myc*, *H-ras*, *c-jun*, and *c-abl*) and tumor suppressor genes (such as *p53*) have also been implicated in the cellular resistance to CDDP [70]. One such example is *c-myc*, an oncogene with the important role of cell proliferation controller that was amplified and over-expressed in several cancer types. Moreover, it can promote cell transformation and induce metastatic features as well as resistance in response to antineoplastic agents [72]. In addition it has been hypothesized that oxaliplatin has a different spectrum of activity and mechanisms of action and resistance, compared to other platinum-containing compounds [60].

While the knowledge of the mechanism of action of CDDP has improved our understanding of resistance [70], the mechanism of decreased intracellular accumulation of CDDP is not well understood. The cell partially controls whether CDDP enters the cell, therefore CDDP does not enter the cell by passive mechanisms (e.g., diffusion) only, but there is an active transport system involved [73]. However, different active uptake and efflux mechanisms are involved, and altered regulation of these transporters could be responsible for the reduced accumulation of the drug in resistant cells. The intracellular mechanisms of acquired resistance to CDDP are: (a) increased detoxification of drugs by the thiols glutathione and metallothionein; (b) improved repair of, and tolerance to, nuclear lesions, leading to a concomitant reduction in apoptosis; and (c) diminished accumulation of cisplatin [73].

The presence of the CDDP adducts in DNA is thought to trigger cell cycle arrest and apoptosis. Decreased intracellular concentration, due to decreased drug uptake, increased reflux or increased inactivation by sulfhydryl molecules, such as glutathione, can cause resistance to cisplatin. Increased excision of adducts from DNA by repair pathways or increased lesion bypass can also result in resistance. Finally, altered expression of regulatory proteins involved in signal transduction pathways that control the apoptotic pathway can additionally affect the sensitivity [70].

CDDP enters the cells and its chloride ligands are replaced by water, forming aquated species that react with nucleophilic sites in cellular macromolecules [70]. Once inside the cell, it interacts with a variety of other molecules besides DNA including sulphur-containing macromolecules, e.g., metallothionein/ glutathione, that sequester cisplatin and remove it from the cell. Metallothionein (MT) is most likely involved in the detoxification of heavy metal ions. MT may contribute to cisplatin resistance. In some cases, the levels of MT are higher in cisplatin-resistant cells, but in other cases, the MT levels are unaffected [70]. Glutathione (GSH) is also involved in detoxification. GSH reacts with the harmful byproducts of aerobic life hydrogen peroxide and organic peroxides. GSH is also essential for maintaining the normal structure of red blood cells. In the presence of cisplatin, GSH forms a GSH-platinum complex that is then eliminated from the cell. The levels of GSH are increased in some—but not all—cisplatin-resistant cells, suggesting that there are other mechanisms of cellular resistance [70].

Alternative ways in which cancer cells might become resistant to cisplatin are based on their ability to remove cisplatin-DNA adducts and to repair cisplatin-induced lesions in DNA. Such abilities might result from the presence of certain DNA repair proteins (e.g., the product of XRCC1 gene; YB1; *etc.*) [30,69]. Studies showed an increase in the levels of DNA repair proteins as tumor cells become less sensitive—and therefore more resistant—to the treatment with CDDP. This suggests that DNA repair is one mechanism which is activated at the very beginning in CDDP resistance. Over time other mechanisms, such as decreased intracellular accumulation and sequestration of CDDP by sulfur-containing macromolecules, may also become significant. CDDP cross-links to DNA, forming intra-and inter-strand adducts, which attract high-mobility-group domain and other proteins. Due to a shielding effect caused by these proteins, the CDDP-modified DNA is poorly repaired, resulting DNA damage triggers cell-cycle arrest and apoptosis [28].

Furthermore, epigenetic modifications could also be another important mechanism of drug resistance. Recent studies have demonstrated that drug-induced alterations in DNA methylation play an important role in this process. The reversal of resistance to CDDP in murine neuroblastoma was induced by inhibition of DNA methyltransferase activity. Thus, DNA methylation plays a central role in onset of drug resistance in cisplatin resistant neuroblastoma cells *in vitro* [74].

Drug-resistance may be also linked to drug-induced dysregulation of microRNA function. Furthermore, evidence indicates the existence of similarities between drug-resistant and metastatic cancer cells in terms of resistance to apoptosis and enhanced invasiveness. Studying the role of miRNA alterations in the acquisition of cisplatin-resistant phenotype in MCF-7 human breast adenocarcinoma cells revealed a total of 103 miRNAs whose expression was changed (46 up-regulated and 57 down-regulated). These differentially expressed miRNAs are involved in the control of cell signaling, cell survival, DNA methylation and invasiveness. The most significantly dysregulated miRNAs were miR-146a, miR-10a, miR-221/222, miR-345, miR-200b and miR-200c. Pogribny and colleagues recently demonstrated that miR-345 and miR-7 target the human multidrug resistance-associated protein 1, suggesting that dysregulation of miRNA expression may underlie the abnormal functioning of critical cellular processes associated with the cisplatin-resistant phenotype [75].

Righetti and his team investigated the gene expression profile of ovarian carcinoma cells exhibiting sensitivity or resistance to platinum compounds. The major substrate of protein kinase C (PKC), and the myristoylated alanine-rich C kinase substrate (MARCKS) were up regulated in drug resistant cells. Unfortunately, its regulatory function has not yet been demonstrated in *in vitro* studies. Therefore, the development of resistance to platinum compounds does not support a general role of PKC as a determinant of the resistance status [71].

The activation of nuclear factor kappaB (NF-kappaB) contributes to drug resistance in pancreatic carcinoma and it was demonstrated that pretreatment of pancreatic carcinoma cells with genistein down-regulates NF-kappaB activity and contributes toward enhancing the apoptosis-inducing effect of cisplatin, leading to greater antitumor activity *in vivo* [76].

Cancers can also become resistant to multiple drugs: *Multi Drug Resistance* (MDR) (Figure 2(8)). This normally results from over-expression of P-glycoprotein (Pgp) and multidrug resistance protein (MRP or MRP1) that function as ATP-dependent efflux pumps. The multidrug resistance, (MDR)-reversing agents, cyclosporin A and PAK-104P, have proven able to reverse the drug resistance to CDDP in some *in vitro* models [77].

## 5. Conclusion

Cancer chemoprevention employs agents that block, hinder, or reverse tumorigenesis to prevent malignancy [41]. CDDP has proven to be one of the more efficient anticancer chemotherapeutic agents because it targets multiple intracellular sites in order to induce death in tumor cells. Nevertheless in order to increase the efficiency of CDDP, other drugs are used in combination. More research is necessary in order to improve anticancer treatments and to decrease toxic effects.
